# Diagnostic accuracy of procalcitonin in proven and clinically suspected systemic infection

**DOI:** 10.1186/cc11693

**Published:** 2012-11-14

**Authors:** S Das, D Anand, S Ray, S Bhargava, A Manocha, M Kankra, LM Srivastava

**Affiliations:** 1 Sir Ganga Ram Hospital, New Delhi, India

## Background

Sepsis is a common cause of morbidity and mortality in critically ill patients. Microbiological culture is the gold standard for diagnosis of sepsis but unfortunately culture results are positive in only 30 to 50% patients. Sepsis is also difficult to distinguish from systemic inflammatory response syndrome (SIRS) because of similar clinical presentations. Procalcitonin (PCT) and IL-6 are known biochemical markers for diagnosis and prognosis of sepsis. The aim of this study was to evaluate the diagnostic role of PCT and IL-6 in differentiating sepsis (both culture positive and culture negative) from SIRS.

## Methods

The study comprised three patient groups, age >18 years: group 1 (*n *= 41), proven infection; group 2 (*n *= 29), clinically suspected infection but negative culture; group 3 (*n *= 29), patients with SIRS. Blood was collected at the time of admission for microbiological culture and estimation of PCT (TRACE, Kryptor) and IL-6 (CLIA, Access).

## Results

The median PCT level was significantly higher (*P *< 0.001) in both groups 1 and 2 as compared with group 3, whereas the median IL-6 level was significantly high (*P *< 0.001) only in group 1 as compared with group 3. Receiver operating characteristic (ROC) curve analysis between groups 1 and 3 for prediction of systemic infection demonstrates that both PCT and IL-6 have a significant area under the curve (AUC) of 0.923 (*P *< 0.001) and 0.824 (*P *< 0.001) respectively. The best cutoff point of PCT was at 1.48 ng/ml with 85% sensitivity, 83% specificity, 88% positive predictive value (PPV) and 80% negative predictive value (NPV). However, the best cutoff point for IL-6 was very high at 219 pg/ml, with 78% sensitivity, 76% specificity, 82% PPV and 71% NPV. Similarly, ROC curve analysis between groups 2 and 3 demonstrates that PCT has a significant AUC of 0.848 (*P *= 0.001), whereas the AUC of IL-6 is 0.555, which is not significant (*P *= 0.47). The best cutoff point for PCT was at 1.45 ng/ml with 83% sensitivity, 79% specificity, 80% PPV and 82% NPV, whereas the best cutoff point of IL-6 was 98.25 pg/ml, with only 59% sensitivity, 55% specificity, 57% PPV and 57% NPV.

## Conclusion

In differentiating SIRS from sepsis, IL-6 does not have a diagnostic role in culture negative sepsis patients, whereas PCT showed a better accuracy in differentiating SIRS from both proven and suspected sepsis. Hence, inclusion of PCT in the initial diagnostic strategy may aid in early and appropriate therapeutic intervention in culture-negative sepsis patients.

